# A review for navigating the trade-offs: evaluating open-source and proprietary large language models for clinical and biomedical information extraction

**DOI:** 10.3389/fdgth.2026.1778786

**Published:** 2026-04-01

**Authors:** Yutaka Sugihara, Aleksandar Milosavljevic, Skaidre Jankovskaja, Magnus Falk

**Affiliations:** 1 Department of Biomedical Science, Faculty of Health and Society, Malmö University, Malmö, Sweden; 2 Biofilms-Research Center for Biointerfaces, Malmö University, Malmö, Sweden; 3 Department of Periodontology, Faculty of Odontology, Malmö University, Malmö, Sweden

**Keywords:** healthcare AI, information extraction, large language models, open-source models, proprietary models

## Abstract

The exponential growth of biomedical data necessitates advanced tools for efficient information extraction (IE) to support clinical decision-making and research. Large language models (LLMs) have emerged as transformative solutions, yet their application in healthcare raises critical trade-offs between open-source (OSS) and proprietary models. This review evaluates IE workflows such as named entity recognition, relation extraction, and terminology normalization, through five axes: performance (including schema fidelity), reproducibility, cost, transparency & auditability, and patient-centric governance. While proprietary models excel in schema compliance and complex reasoning, OSS models offer advantages in auditability, local control, and cost-effectiveness. Challenges such as schema fidelity, reproducibility, and ethical considerations like algorithmic fairness and data sovereignty are emphasized. The analysis highlights that OSS models, though requiring domain-specific adaptation, enable greater transparency and customization for privacy-sensitive tasks, whereas proprietary systems face limitations in bias mitigation and regulatory alignment. By addressing technical, ethical, and operational challenges, this work underscores the importance of context-aware model selection to balance innovation with accountability in clinical AI deployment. The findings advocate for hybrid approaches that integrate OSS flexibility with proprietary capabilities, ensuring equitable, reliable, and compliant healthcare solutions.

## 1 Introduction

The rapid expansion in scientific literature, clinical records, and complex biomedical information presents both a significant opportunity and a substantial challenge (1). While this data holds immense potential for advancing our understanding of health and disease, traditional methods are ill-equipped to handle large volumes and complexity. The sheer scale and heterogeneity of biomedical data, spanning diverse formats, languages, and domains, further complicate efforts to extract meaningful insights, underscoring the urgent need for more adaptive and scalable analytical tools (2, 3).

Large language models (LLMs), a class of artificial intelligence (AI) algorithms capable of comprehending and generating human-like text, offer a promising solution to this challenge (4, 5). Trained on extensive datasets comprising text, code, and structured data, LLMs show promise in tasks such as document summarization, language translation, and identifying complex patterns in diverse data (6). LLMs have shown particular promise in tasks such as literature review (7), information extraction (IE) (8, 9), and supporting data-driven analysis (10). By efficiently processing vast amounts of medical documents and/or literature, LLMs can help identify novel patterns and correlations (11, 12). Their remarkable text comprehension capabilities and impressive generality allow them to adapt to diverse biomedical tasks and datasets. This could eventually lead to groundbreaking discoveries in several domains such as disease mechanisms, personalized treatments, and drug development. Hence, LLMs could have significant potential to improve biomedical research and clinical practice (13, 14).

Structuring and standardizing medical data is crucial for improving diagnostic accuracy and enabling patient data sharing and integration. IE from medical documents and organizing it according to standardized schemas like Fast Healthcare Interoperability Resources (FHIR), a standard for exchanging healthcare data between systems, serves as an ideal filter for evaluating the capabilities of LLMs. IE particularly tests the ability to standardize medical terminology (e.g., mapping to SNOMED-CT and/or ICD-10) and directly raises concerns about data transparency and auditability. This allows for a deep exploration of how the choice between open-source (OSS) and proprietary models impacts clinical workflows, patient equity, and trust. Furthermore, because IE results can directly impact diagnosis and treatment, patient-centric fairness and data reliability are essential evaluation criteria.

The existing literature on IE in healthcare lacks critical scrutiny of two underexplored dimensions. First, the trade-offs between OSS and proprietary LLMs are underexplored. While performance metrics dominate discussions, few reviews have explicitly addressed the implications of model choice on reproducibility, transparency, cost, regulatory compliance, and sustainability in clinical workflows. Specifically, the limitations of proprietary LLMs are exemplified by a lack of auditability, hindering efforts to identify and mitigate potential biases (15, 16). Conversely, open-source LLMs face limitations in domain adaptation, making it challenging to tailor them effectively to the nuances of healthcare practice. Second, the patients' perspective is visibly absent. Issues such as algorithmic fairness (e.g., underrepresented groups in training data leading to disparities in diagnostic accuracy), explainability for patient trust, and data sovereignty (e.g., risks of sensitive data exposure in proprietary systems) are rarely integrated into evaluations (17–20). These omissions risk maintaining disparities and weakening public confidence in AI-driven healthcare solutions.

While our review covers multiple clinical NLP tasks, we focus on IE as the most illustrative lens for comparing OSS and proprietary models. IE uniquely emphasizes schema-constrained generation (e.g., FHIR), Protected Health Information (PHI) handling, normalization to clinical terminologies, and end-to-end auditability. This review therefore provides a patient-governed comparison across five axes: performance (including fidelity), reproducibility, cost, transparency & auditability, and patient-centric governance.

## 2 Technical background

The technical foundations discussed in this section directly support IE workflows. Named entity recognition (NER), term normalization, assertion status detection, relation extraction (RE), and schema-based structuring are key components. Each of these components is evaluated based on axes such as schema fidelity, reproducibility, and auditability. These tasks collectively expose failure modes (e.g., hallucinated entities, incorrect negation handling, normalization mismatches) that strongly influence OSS vs. proprietary trade-offs. These foundations matter most when IE outputs must satisfy strict schemas and normalization, which is precisely where open-source vs. proprietary trade-offs become significant (Table 1).

**Table 1:** Representative IE tasks and evaluation axes.

IE Task	Technical Requirement	Evaluation Axis	OSS Advantage	Proprietary Advantage	LLMs	References
NER	Entity extraction	Performance, reproducibility	Local control, reproducibility	High accuracy, minimal tuning	GPT-3.5 Turbo, GPT-4 Turbo, Llama-2-13b-hf	Luo et al. (2023) (8), Dietrich et al. (2025) (67)
Assertion Status Detection	Negation & certainty handling	Performance; Transparency & Auditability	Log acquisition possible	High accuracy	GatorTron-medium	Tay et al. (2024) (29)
RE	Relation extraction	Performance (Schema fidelity)	Flexible customization	Complex reasoning	Taiyi, T5	Luo et al. (2023) (8), Diaz-Garcia et al. (2025) (23)
Term Normalization	Terminology standardization	Performance (Interoperability)	Dictionary control possible	High-accuracy mapping	GPT-4	Li et al. (2024) (93), Rossander et al. (2021) (36)
Schema-based structuring	Structured transformation	Performance (Schema fidelity), Transparency & Auditability	Local validation possible	High format compliance	GPT-4o, Gemini-2-Flash, Llama-3.1-8B-Instruct	Idrissi-Yaghir et al. (2025) (38), Xia et al. (2024) (37)

### 2.1 Information extraction fundamentals

LLMs have emerged as transformative tools for converting unstructured biomedical data into structured formats, enabling seamless communication between humans and machines through standardized data representations such as JSON, CSV/TSV, or Markdown. This capability is particularly critical in healthcare, where clinical notes, radiology reports, and other textual data often lack standardized structures. By automating the extraction of structured information, LLMs reduce the labor-intensive manual processes traditionally required for data annotation and interpretation, thereby accelerating downstream tasks such as clinical decision support, research analysis, and electronic health record (EHR) integration.

In addition to IE, LLMs are also being investigated for summarization in clinical settings, where the ability to condense large volumes of unstructured data into concise, actionable summaries is essential (21). For example, summarization can help clinicians quickly review patient histories, extract key findings from radiology reports, or generate structured summaries for EHRs. This task is particularly valuable in resource-constrained environments, where time and efficiency are critical. However, summarization differs from information extraction in that it focuses on condensing and rephrasing rather than extracting structured fields. Both tasks are complementary and contribute to the broader goal of enhancing data usability in healthcare.

Two representative tasks support the application of LLMs in IE: NER and RE. NER involves identifying and classifying entities within text, such as diseases, medications, anatomical terms, or genetic markers, while RE focuses on uncovering semantic relationships between these entities (e.g., “Drug X treats Disease Y”). Traditional IE approaches rely on supervised learning, where models like BERT (Bidirectional Encoder Representations from Transformers, a neural network that learns word meaning from both left and right context) are fine-tuned on manually labeled datasets to achieve high accuracy (22). For instance, a sentence like “The patient's temperature is 37 degrees” might be annotated with “patient” as a PERSON entity and “temperature” as a MEASUREMENT, while a relationship such as (“patient”, “has”, “temperature”) could be extracted for RE (23). However, these methods require extensive labeled data, which is often scarce or costly to generate in healthcare settings.

In contrast, LLMs leverage contextual learning to perform NER and RE with minimal supervision, significantly reducing the dependency on large annotated datasets (6, 24, 25). For example, models like GPT-4 can infer entity types and relationships from sparse examples, making them particularly suitable for domains with limited labeled data, such as rare diseases or niche clinical specialties (26–28). This flexibility is clearly demonstrated by a recent system that identifies metastatic disease in radiology reports from diverse primary cancer types (29). This system employs a pipeline architecture comprising four key components:

1. Named Entity Recognition (NER): Identifies and classifies entities (e.g., “lung” as a body part).
2. Term Normalization: Standardizes terminology (e.g., mapping “lung cancer” to “malignant neoplasm of lung”).
3. Assertion Status Detection: Determines the certainty of entities (e.g., “no evidence of metastasis”).
4. Relation Extraction (RE): Establishes semantic links between entities (e.g., “tumor in lung”).

While NER and RE are foundational to general IE, the inclusion of Assertion Status Detection and Term Normalization reflects domain-specific requirements in medical contexts. This addresses challenges like negation handling, interoperability, and terminology ambiguity (30, 31). These are categorized as essential for medical IE pipelines but not core to general NLP-based extraction tasks.

By integrating these steps, the system achieves high accuracy in inferring metastatic sites across diverse cancer types, with F1 scores ranging from 0.89 to 0.96 for individual cancers (F1 = harmonic mean of precision and recall; closer to 1 is better) (29). These results underscore the potential of LLMs to extract and structure clinically actionable information from unstructured radiology reports, bridging the gap between raw text and structured data.

Beyond radiology, LLMs have demonstrated strong performance in domain-specific tasks such as medication extraction from clinical notes (32). Studies highlight that adapting LLMs to domain-specific data, through methods like prompt engineering, few-shot learning, or domain-specific fine-tuning, enhances their performance in tasks such as classification (6), information extraction (6, 8–10), and evidence synthesis (33). This adaptability positions LLMs as multifunctional tools for biomedical IE, capable of addressing the complexity and variability of clinical data. However, without consistent terminology, extracting structured data from diverse sources leads to ambiguity and inaccuracies. This necessitates term normalization to ensure the reliability of structured data.

Term normalization is a critical process for ensuring data consistency and reliability across diverse healthcare data sources. While traditional approaches often rely on manual curation or rule-based systems to map heterogeneous terms (e.g., “lung cancer” corresponds to “malignant neoplasm of lung”), LLMs offer a scalable and context-aware alternative. By leveraging their pre-trained knowledge of biomedical terminology and contextual understanding, LLMs can automatically infer standardized representations for entities, even when input terms are ambiguous or vary across institutions. For instance, a model like GPT-4 can map “lung carcinoma” and “pulmonary tumor” to a unified code (e.g., SNOMED CT or ICD-10) without explicit supervision, reducing the need for labor-intensive manual normalization (34, 35). These procedures ensure the reusability and auditability of IE outputs, thereby enhancing to accountability towards patients.

This capability is particularly valuable in biomedical IE, where term normalization directly impacts the interoperability of data across EHRs, research databases, and clinical decision-support systems. By integrating term normalization into their pipeline, LLMs not only enhance the consistency of extracted entities but also improve the downstream utility of structured data. For example, in the metastasis detection system described earlier, term normalization ensures that “lung” and “pulmonary region” are treated as equivalent, enabling accurate relation extraction and statistical analysis. Such integration aligns with the broader goal of LLMs to minimize reliance on annotated datasets while maximizing the accuracy and generalizability of biomedical IE tasks.

Although IE is important for clinical interoperability and research acceleration, it has not yet become a dominant workload in real-world LLM deployments. This is due to several factors: (i) technical complexity, including strict schema compliance (e.g., FHIR), accurate negation handling, and normalization to terminologies such as SNOMED CT or ICD-10 (34, 36); (ii) lack of standardized benchmarks beyond emerging efforts like FoFo that is Format-Following Benchmark, which tests how well LLMs generate outputs that conform to specific schema formats and Note2FHIR that is a framework that transforms clinical notes into structured FHIR (37, 38); and (iii) regulatory and ethical constraints, such as PHI protection, auditability, and reproducibility requirements. These challenges explain why IE adoption lags behind summarization and QA, even though it offers the most direct pathway to structured, interoperable clinical data.

### 2.2 LLM evolution and application to IE

While OSS and proprietary LLMs differ in deployment and licensing, their comparative strengths become most evident in IE tasks. OSS offers auditability and local control, critical for PHI-sensitive extraction, whereas proprietary systems often excel in complex reasoning and strict schema compliance.

Further fine-tuning and evaluation of LLMs leads to broader applicability and improved performance (9, 39). A common pattern emerges where fine-tuning LLMs for domain-specific tasks improve their performance (29, 40). This indicates that adapting LLMs to specific domains, such as healthcare and/or biomedicine, is crucial for achieving optimal results. However, the limitations of fine-tuning alone, such as the need for extensive labeled data and the risk of overfitting, highlights the importance of complementary approaches. For instance, meticulous data collection and standardization practices are crucial for realizing the full potential of LLMs, as high-quality datasets directly correlate with model performance (41, 42). The creation of robust datasets relies on both the curation of diverse (43) and representative data and the adoption of standardized annotation frameworks (44).

The effectiveness of LLMs in interpreting and processing clinical information depends both on the quality of their training data and on the standardization of the clinical text provided at inference time. In practice, inconsistencies and ambiguities in unstructured clinical note, such as variable phrasing like “heart attack’ or “myocardial infarction”, can degrade performance, making robust data normalization essential for accurate interpretation. Specifically, the adoption of standardized terminologies like SNOMED CT and ICD-10 is critical (45, 46). SNOMED CT, with its comprehensive, multilingual clinical healthcare terminology, allows for precise representation of clinical findings, diseases, procedures, and more. Mapping unstructured clinical notes to SNOMED CT concepts facilitates semantic understanding by the LLM, enabling more accurate information retrieval and analysis. Similarly, ICD-10 provides a standardized coding system for diagnoses and procedures, allowing for consistent data aggregation and comparison across different healthcare settings. For example, instead of relying on variable phrasing like “heart attack” or “myocardial infarction,” mapping these terms to the standardized SNOMED CT code 22298006 (Acute myocardial infarction) ensures that the LLM consistently interprets these concepts (36, 47).

In addition to directly prompting LLMs to generate SNOMED codes, another effective approach is Retrieval-Augmented Generation (RAG). RAG retrieves relevant information from a vector database based on the query and incorporates it into the LLM's input to generate more accurate SNOMED codes. While fine-tuning offers the advantage of reduced dependency on external databases, RAG excels in its ability to integrate the latest clinical information. However, this strength also introduces risks: if the underlying database is outdated or contains inaccurate data, RAG may generate incorrect SNOMED codes. This reliance on external data underscores the critical role of standardization, not only as a means to ensure consistent data representation, but also as foundation for methods like RAG to function reliably. Therefore, standardization not only improves model accuracy but also facilitates interoperability and data sharing.

Beyond individual techniques, researchers have investigated the synergistic potential of combining methods like instruction tuning, multi-task learning, and fine-tuning to achieve enhanced LLM performance (48, 49). For example, instruction tuning enables models to follow complex, task-specific instructions, while multi-task learning allows them to generalize across related tasks. This hybrid approach may offer additional benefits, such as improved robustness and reduced dependency on large, labeled datasets. Combining instruction tuning and fine-tuning allows LLMs to both follow complex task instructions and acquire specific domain knowledge (50, 51). For example, Instruction tuning is first employed to train the model task formatting, followed by fine-tuning to optimize its representation of medical text. Future effort should explore these combinations, particularly focusing on healthcare settings where domain-specific challenges require tailored solutions (50, 52, 53).

While standardization practices are critical for general LLM applications, they face unique challenges in specialized domains like rare diseases. A significant limitation in applying LLMs to rare diseases is the scarcity of labeled training data. Rare diseases, by definition, affect a small percentage of the population, resulting in limited clinical records and research publications. This data limitation can significantly hinder the performance of LLMs, leading to inaccurate predictions and unreliable insights (54, 55). To address this issue, various approaches can be employed. Few-shot learning and transfer learning techniques allow LLMs to generalize from limited examples by leveraging knowledge gained from related tasks or domains (56, 57). For example, an LLM trained on common cardiovascular diseases can be fine-tuned on a small dataset of patients with a specific rare cardiomyopathy. Furthermore, knowledge injection techniques, where explicit domain knowledge is incorporated into the LLM, can compensate for data scarcity. This can be achieved through the integration of specialized medical ontologies, curated databases of rare disease information, and expert-derived clinical guidelines (58, 59). Finally, Generative Adversarial Networks (GANs) and other synthetic data generation methods can address the limited dataset by creating realistic, artificial patient records. However, careful validation is crucial to ensure the quality and representativeness of the synthetic data (60). This has the potential to reduce errors in interpreting negation and medical terminology, especially when applying IE to rare diseases, but inherent limitations of IE still exist.

In short, IE faces persistent challenges such as accurately handling negation and uncertainty (e.g., distinguishing between “patient denies chest pain” and “patient reports chest pain”), identifying correct relationships between entities (e.g., associating a medication with the correct patient), and ensuring consistent normalization of clinical terms (e.g., mapping “heart attack” and “myocardial infarction” to a single standardized concept). These failure modes directly impact the trade-offs between schema reliability, auditability, and cost when deploying LLMs in clinical settings.

### 2.3 Healthcare-specific challenges

Fairness challenges in LLM-based information extraction arise when NER or RE accuracy varies systematically across demographic, linguistic, or contextual subgroups. For instance, models may exhibit lower precision for rare entity types (e.g., uncommon medical conditions) or underperform on underrepresented linguistic groups due to imbalanced training data. Mitigation approaches include domain-specific data augmentation, fairness-constrained training objectives, and subgroup-specific evaluation metrics to ensure equitable extraction performance across diverse populations.

Medical data inherently exhibit unique complexities that challenge the applicability of standard fairness metrics. For instance, comorbidities, genetic backgrounds, and regional disparities introduce domain-specific covariates that are often confounding factors in healthcare outcomes. These factors may exhibit complex non-linear relationships with model predictions, rendering traditional fairness metrics, such as demographic parity or equalized odds, insufficient to capture the nuanced relationships between model decisions and patient-specific contexts (61). Addressing these biases is crucial for ethical and effective LLM deployment in healthcare. Researchers emphasize the need for proactive bias mitigation strategies (42, 62). These bias mitigation techniques include (1) curating diverse training datasets, (2) implementing fairness metrics, and (3) rigorous bias testing. These techniques, such as employing fairness metrics, domain-specific fine-tuning, and data standardization, align with the technical approaches discussed in Section 2.2, particularly domain-specific fine-tuning and data standardization are critical for bias reduction. Ensuring that training data encompasses diversity, particularly in terms of racial representation, and transparently communicating such diversity to end users can increase expectations of algorithmic fairness and trust in AI, thereby helping to mitigate the reproduction and amplification of existing biases (63). During model training, incorporating fairness metrics that measure potential bias against protected groups can guide the model towards more equitable outcomes (64, 65). Conducting thorough testing on diverse patient populations can help identify and address any remaining biases before deploying the LLM in real-world settings (66). Consequently, promoting data diversity and rigorous assessment of model fairness are essential to safeguard patient equity.

## 3 OSS vs. proprietary models

This section compares open-source and proprietary LLMs across clinical NLP tasks broadly, while highlighting IE as a representative workload for understanding trade-offs in performance (including schema fidelity), reproducibility, cost, transparency & auditability, and patient-centric governance (Table 2). When relevant, we anchor metrics and examples in IE sub-tasks (NER, term normalization, assertion status detection, and RE) to make operational implications concrete.

**Table 2:** Trade-offs between OSS and proprietary LLMs across five critical dimensions for clinical IE.

Axis	Open-Source (On-Premise) LLMs	Proprietary (Cloud/API) LLMs
Performance (Schema Fidelity)	Moderate to high, but dependent on local model choice and tuning; requires in-house optimization to reach top performance levels	Typically high due to vendor-optimized architectures and large-scale training pipelines
Reproducibility	Strong: model weights, prompts, and environments can be fully preserved and version-controlled, enabling reproducible workflows	Limited: proprietary models evolve continuously, often without version-level transparency, making strict reproducibility challenging
Cost	High: inference cost is generally low once infrastructure is in place; predictable operational expenses	Variable: usage-based pricing can become expensive at scale; cost efficiency improves only with very large volumes or enterprise contracts
Transparency & Auditability	High: model internals, logs, and inference processes are inspectable; supports full auditing and bias analysis	Low to moderate: internal model mechanisms and logs are restricted, limiting explainability and detailed audits
Patient-Centric Governance	Strong alignment possible, because deployment is fully controlled by the institution; policies can be strictly customized to local governance and ethics requirements	Governance depends on provider policies; customization is limited, and data governance relies on contractual agreements and service compliance

### 3.1 Definition and deployment models

The distinction between OSS and proprietary LLMs is central to understanding their role in clinical IE. OSS models provide unrestricted access to model weights and source code, enabling institutions to deploy models locally, perform domain-specific fine-tuning, and maintain comprehensive audit trails. These capabilities align with regulatory imperatives under frameworks such as GDPR and HIPAA, which prioritize data sovereignty and transparency (67–70). OSS transparency also facilitates reproducibility and bias auditing, allowing healthcare organizations to interrogate model behavior and implement explainability frameworks that strengthen clinicians’ confidence (71–73).

Proprietary models, by contrast, are typically accessed via cloud-based Application Programming Interface (API), offering immediate access to frontier performance and advanced capabilities such as multimodal reasoning and strict schema adherence (74–76). These benefits, however, come at the cost of transparency and reproducibility, as internal weights and training data remain inaccessible to end-users (77). This lack of transparency complicates bias detection and accountability in high-stakes workflows, creating conflict between performance optimization and ethical governance (61). Licensing restrictions further worsen these trade-offs. While OSS frameworks grant freedom to modify and distribute, proprietary systems retain vendor control, limiting customization and local validation (78). This vendor control also raises concerns about long-term dependency and vender reliance, as institutions may face challenges in migrating to alternative systems or adapting to evolving regulatory requirements. For example, proprietary models often require adherence to specific licensing terms, which can restrict the use of model outputs in certain clinical workflows or limit integration with legacy systems. These factors further demonstrate the necessity of hybrid architectures, which implement a two-tier approach: local retrieval and embedding (hosted on-premises) for PHI management and audit trails, coupled with cloud-based generation (e.g., GPT-4o) for high-accuracy clinical inference. This design enables institutions to satisfy regulatory mandates like HIPAA while leveraging frontier model capabilities.

Deployment models highlight these differences. On-premises OSS can keep PHI within institutional networks. In a multi-hospital study, locally run Gemma2-9B-instruct achieved an aggregate F1 ≈ 0.965 for echocardiography information extraction, although the authors note occasional hallucinations and the need for validation and post-processing (68). This configuration not only preserved privacy but also enabled fine-grained auditability. This is a requirement for regulated environments. Conversely, cloud-hosted proprietary LLMs (e.g., GPT-4/4o) have demonstrated higher accuracy than local open-source models for radiology report error detection across X-ray, ultrasound, CT, and MRI, highlighting their potential for multi-subspecialty QA, though the authors note privacy concerns with cloud processing in clinical practice (79). Hybrid architectures represent a balanced approach, combining local retrieval and embedding with cloud-based generation to balance privacy, performance, and cost. Optimized local RAG pipelines (e.g., LLaMA3 with dense retrieval) achieved total response latencies of ∼1.3–1.7 s, comparable to or faster than GPT-4o's ∼2.7–4.0 s, while keeping institutional documents on-prem via local hosting. However, more advanced retrieval strategies can trade higher accuracy for increased latency (80).

### 3.2 Performance

Performance comparisons between OSS and proprietary LLMs indicate varied trade-offs across clinical IE. Within biomedical RE, domain-adapted pipelines and select OSS LLMs (e.g., DeepSeek-V3) have been reported to outperform proprietary models (GPT-4o/4.1), with performance trade-offs varying widely depending on task design and optimization (81). However, proprietary models tend to outperform OSS on format-following/strict schema-adherence benchmarks (e.g., FoFo), and they achieve near-perfect accuracy on some FHIR knowledge tasks such as resource type identification. In contrast, for end-to-end Note-to-FHIR generation, all models, including proprietary ones, still fall short, with the best reported performance far from perfect compliance (37, 38).

Radiology QA exemplifies these differences. GPT-4o detected 88% of inserted errors, compared with 79% for LLaMA-3-70B and 73% for Mixtral-8 × 22B. In text-based differential diagnosis across radiology subspecialties, GPT-4o also achieved higher accuracy than open-source models (e.g., 79.6% vs. 73.2% for LLaMA-3-70B). However, hallucination rates, especially in multimodal diagnostic tasks, remain substantial and task-dependent (79, 82). OSS models offered latency advantages (≈6 s vs. 13 s per report) and on-prem deployment for PHI compliance, highlighting the operational trade-offs between accuracy and governance.

Similarly, clinical trial matching demonstrates OSS potential for equivalence under targeted adaptation. After fine-tuning on a limited synthetic dataset, Trial-LLAMA-70B matched GPT-3.5 on explicit eligibility criteria, while GPT-4 remained stronger on implicit (unspoken) reasoning tasks (78). These results emphasize that OSS can deliver competitive performance for privacy-sensitive deployments, provided rigorous validation and domain-specific tuning are implemented. Table 3 summarizes the comparative strengths and limitations of open-source and proprietary LLMs across five axes relevant to clinical IE workflows.

**Table 3:** OSS vs. proprietary model comparison in IE tasks.

Evaluation Axis	OSS Models	Proprietary Models	LLMs	References
Performance (including schema fidelity)	Strong NER/RE after tuning; schema-constrained outputs (e.g., FHIR) typically require additional prompt engineering or validation	High out-of-the-box accuracy and reasoning; generally superior format compliance for schema-constrained tasks	Llama 3.1 8B Instruct, Mistral 3 Small 24B Instruct, GPT-4o	Khairat et al. (2025) (84), Sandmann et al. (2025) (85), Kim et al. (2025) (92)
Reproducibility	High: local execution, determinism, version locking, full pipeline re-runs and logging are feasible	Lower: API dependency, model updates outside user control, limited determinism and audit trails	GPT-3.5 Turbo, GPT-4 Turbo, Llama-2-13b-hf	Dietrich et al. (2025) (67)
Cost	Predictable operational costs after initial setup; can optimize via quantization/distillation; favorable at scale	Usage-based fees with potential variability; frontier performance often more expensive; long-term predictability limited	llama2:7b-chat, mistral:7b-instruct, GPT-4	Irugalbandara et al. (2024) (91), Adams et al. (2025) (90)
Transparency & Auditability	High: weights/source visible; easier bias audits, evidence tracing, and explainability; granular logs	Low: black-box internals; audits rely on vendor disclosures and external safeguards; limited visibility into model behavior	GPT-4	GDPR Article 5 (69), Gallifant et al., (2024) (77), Rosenbacke et al., (2024) (72)
Patient-Centric Governance	Strong: on-prem deployment enables data sovereignty and GDPR/HIPAA alignment; fairness and multilingual tuning under local control; supports human-in-the-loop	Weaker: cloud/data transfer considerations; governance relies on contracts and external audits; fairness/multilingual safeguards may be less customizable	Gemma2:9b-instruct, Llama3:70b, GPT-4o	Chi et al. (2025) (68), Salam et al. (2025) (79)

Schema fidelity is critical in clinical IE because outputs often feed directly into EHR systems and decision-support tools. Tasks such as converting unstructured notes into FHIR resources require strict adherence to predefined schemas and accurate normalization to terminologies like SNOMED CT and ICD-10 (36), otherwise, errors can lead to incorrect diagnoses and treatment plans. Proprietary models tend to show stronger out-of-the-box adherence to prescribed formats than OSS models. In domain-specific schemas such as FHIR, however, even top models struggle in zero-shot generation, indicating the need for additional constraints and validation (37, 38).

These observations should be interpreted as contextual deployment considerations rather than inherent deficiencies of open-source models. With appropriate prompt engineering, retrieval grounding, fine-tuning, and validation layers, open-source systems can substantially narrow performance gaps, although this often requires additional engineering effort in regulated clinical environments.

Hybrid approaches are emerging as a pragmatic solution for clinical IE, particularly in tasks requiring strict schema fidelity such as Note2FHIR conversion. OSS components excel in auditability, reproducibility, and local control, enabling compliance with privacy regulations and facilitating bias audits. However, they often require extensive prompt engineering and domain-specific fine-tuning to achieve schema-constrained outputs. Proprietary models, by contrast, deliver higher accuracy by default and superior adherence to structured formats such as FHIR, but at the expense of transparency and data sovereignty. This trade-off becomes critical in regulated workflows where errors in entity extraction or normalization can propagate into clinical decisions, underscoring the need for architectures that partition tasks to balance accuracy, cost, and governance (37, 38, 67).

A representative hybrid pattern involves using OSS for preprocessing, such as PHI-safe entity extraction and terminology normalization, followed by proprietary generation for schema-constrained structuring. This design mitigates the opacity of closed systems while leveraging their strengths in complex reasoning and format compliance. Studies show that OSS pipelines can achieve near-parity with proprietary models in sub-tasks like NER and RE when fine-tuned (53, 83), while proprietary ecosystems often provide stronger built-in support for strict output validation and are frequently reported to lead on certain multi-step reasoning benchmarks, even though some studies find open-source parity in clinical decision support (84, 85). It is also important to consider the role of common data elements (CDEs) in maintaining schema fidelity. Initiatives such as RadElement, which aim to standardize data elements for radiology reporting (86, 87), exemplify the need for consistent and interoperable schemas. Adherence to CDEs and leveraging initiatives like RadElement can significantly improve the accuracy and reliability of structured output validation and integration with existing healthcare systems (88). These efforts are crucial for ensuring data exchange, facilitating secondary data analysis, and ultimately enhancing the clinical utility of IE outputs. By integrating local auditability with cloud-based performance, hybrid strategies offer a scalable pathway to meet clinical requirements for transparency, fairness, and interoperability without incurring prohibitive costs or compromising patient trust.

### 3.3 Reproducibility

LLMs utilize non-deterministic algorithms, meaning their outputs are not fixed even with the same input. Factors such as the temperature parameter and random seed influence the generated text. Furthermore, controlling reproducibility is only possible in some LLM implementations, as not all providers expose parameters such as random seed control or fully deterministic decoding options (67). This lack of reproducibility is a major challenge for using LLMs in regulated or controlled environments, such as pharmaceutical pharmacovigilance (GxP), where consistent results are a prerequisite for system validation (67). While this characteristic is beneficial for tasks requiring creativity, it introduces challenges for IE tasks, where consistent and reproducible results are paramount. These include: (1) local execution of the model, utilizing OSS deployed on-premise; (2) strict version locking of the model weights to prevent unintended changes; and (3) comprehensive logging of all relevant experimental parameters, including the input prompts, token usage, and timestamps. These methods provide the necessary control to reproduce our results with a specific configuration.

Despite the introduction of “seed” parameters and “system fingerprints” to improve consistency, studies found that these models still failed to produce reproducible results (67). Experiments showed that a single “system fingerprint” could generate different responses, and different fingerprints could produce the same result, making their use difficult in GxP-validated systems (67). On the other hand, locally hosted open-source models, such as Zephyr-7b-beta or Llama-3 7B, demonstrated significantly higher reproducibility (67). Because they are run on local hardware without the unpredictable parallel execution found in distributed cloud architectures, their random number generators can more reliably be seeded to substantially reduce variability and, under controlled configurations, achieve near-deterministic behavior (67).

Reproducibility is often measured as “variance” or change over time across multiple runs (89). Interestingly, techniques like Chain-of-Thought (CoT) prompting can improve accuracy. However, they do not necessarily decrease the variance or stabilize the model's performance across different iterations (89).

To ensure reproducible evaluation, researchers recommend conducting multiple experimental repeats (often three or more) and using prediction intervals to quantify the uncertainty of benchmark scores (67). It is also considered essential to document the precise API version, model parameters, and date of the experiment.

### 3.4 Cost

IE tasks amplify cost and latency sensitivity due to long-context processing and repeated schema checks. Proprietary cloud APIs often lead to high per-token costs and unpredictable latency, particularly for large-scale clinical QA with long documents. For example, in a recent benchmark, projected expenses reached ≈$8,515 for a single evaluation configuration at 16k-token inputs, leading to the exclusion of a frontier model (90). OSS models, while requiring local infrastructure, offer more predictable costs and have demonstrated 5–29× lower token costs than proprietary APIs while maintaining competitive accuracy for structured IE (91). Hybrid deployments using RAG and Small Language Models (SLMs) further improve affordability and reduce network-induced latency variability, making them attractive for privacy-sensitive, high-volume workflows. However, cost-driven shortcuts, such as skipping fine-tuning or relying on synthetic data without validation, pose ethical risks, including reduced accuracy in critical tasks and fairness gaps (79, 92). Consequently, regulatory frameworks increasingly link fairness and transparency to compliance, making cost-driven compromises a potential institutional risk (61).

An additional consideration is cost predictability over time. Proprietary LLM services are typically offered through usage-based pricing models that may change as providers adjust infrastructure, scaling strategies, or business models. While costs may decrease with technological advances, they may also increase, and institutions have limited control over future pricing or service availability. In contrast, OSS deployed locally offer greater cost predictability, as compute, storage, and maintenance expenses are borne directly by the institution and can be planned and budgeted over longer time horizons.

### 3.5 Explainability: transparency & auditability

Transparency and auditability are crucial elements of reliable clinical IE. Transparency refers to the clear exposition of a model's internal structure and the rationale behind its outputs. Auditability is the ability to trace and review the history of outputs and the processes that generated them. Explainability, encompassing a demonstrable understanding of how a model arrives at its outputs, is not only required by regulatory frameworks but also fundamentally supports auditability and fosters confidence among clinicians and patients. In practice, explainability is embedded within the IE pipeline through three complementary mechanisms: evidence linking, structured-output validation, and local behavior logging. While each of these mechanisms can be implemented using either OSS or proprietary models, the degree of control, transparency, and associated costs (e.g., development, maintenance, and infrastructure) can differ significantly.

#### 3.5.1 Evidence linking

Evidence linking attaches each extracted entity or relation to its source text. For example, the phrase “3.2 cm mass in the left lung” in a radiology report is mapped to the exact character offsets or sentence ID. This traceability is essential for clinicians to verify the model's decision and for regulators to audit PHI handling. OSS models allow developers to embed custom provenance fields directly into the output JSON, and to store the provenance locally, which is required by GDPR/HIPAA compliance. Proprietary models often expose a “source-span” field or a “confidence” score, but the granularity is limited and the provenance may be truncated when the data leave the vendor's infrastructure.

#### 3.5.2 Structured-output validation

Even high-performing models can occasionally produce outputs that fail to conform to the target schema. In healthcare contexts, terminology mismatches have been observed in practice, especially when converting unstructured notes into FHIR-compliant resources (38, 93). To mitigate this, open-source pipelines often run a local schema/terminology validator after generation (94). In practice, FHIRPath/invariant checks help ensure required fields are present (95). Terminology validation can verify that SNOMED CT concept codes are used correctly (96). Custom rule engines can enforce local business logic (e.g., “if a diagnosis is malignant, then a treatment plan must be listed”). While proprietary systems may advertise “schema-compliant” outputs, validation is often performed server-side and detailed audit logs may not be exposed to end users (93). By contrast, open-source pipelines, though they typically require more prompt engineering and post-processing, enable a transparent and reproducible validation loop through local execution of the FHIR validator and terminology checks (97).

#### 3.5.3 Behavior logging

Behavior logging can be designed to capture key elements of the inference workflow (e.g., prompts, model/version identifiers, token usage, timestamps, and raw outputs), aligned with organizational policy and regulatory requirements. In OSS deployments, logs can be written to secure, tamper-evident stores or hospital audit services to support transparent, reproducible monitoring loops. Such detailed telemetry can support bias audits and model-drift monitoring in healthcare; effectiveness depends on metrics, tests, and alerting practice (98, 99). For proprietary APIs, audit features are typically available, but log granularity and retention vary by vendor; teams should verify provider documentation and align with governance frameworks. In multilingual settings, OSS models can be fine-tuned on local corpora, and logs can capture language-specific usage to facilitate targeted bias audits (100).

In summary, explainability is not a single feature but a suite of practices that together form a governance tool. OSS models provide the transparency, auditability, and flexibility needed for regulatory compliance and ethical responsibility, while proprietary models offer convenience and, in some cases, higher out-of-the-box accuracy. A hybrid architecture, local evidence linking and validation combined with cloud-based generation, often delivers the better balance for clinical IE.

### 3.6 Patient-centric AI governance

Current implementation frameworks for patient-centric AI governance emphasize actionable strategies that address algorithmic fairness, accessibility, and value integration. Key approaches include hybrid architectures that combine OSS transparency with proprietary performance, enabling auditability and local control while leveraging complex reasoning capabilities (101). Multilingual fine-tuning and bias audits are critical to mitigate subgroup performance disparities, such as reduced diagnostic accuracy for minority populations or increased hallucination rates in non-English contexts (102). These techniques can support equitable access and help reduce health inequities by aligning AI systems with diverse linguistic and cultural needs (103). Human-in-the-loop validation is essential in high-stakes clinical workflows to ensure outputs are reviewed for fairness, accuracy, and alignment with patient values (103). Additionally, explainability tools and user-friendly applications with clear instructions and feedback mechanisms promote patient trust and transparency (101). By embedding patient feedback loops and multilingual guardrails, healthcare professionals can create technically robust systems that reflect patient-centered principles. These strategies provide a practical pathway to balance technical capabilities with ethical considerations, ensuring AI tools meet technical benchmarks while addressing the diverse needs of served populations.

Healthcare AI systems operate under strict regulatory frameworks such as GDPR and HIPAA, which mandate data minimization, transparency, and accountability. Proprietary models used for clinical text applications raise documented concerns around data privacy and operational governance. Recent evidence shows that both OSS and proprietary models are being explored in healthcare, but limited external validation and inconsistent reporting underscore the need for stricter oversight of vendor-controlled environments (22). OSS models deployed on-premises enable full control over PHI (68).

Data sovereignty is increasingly recognized as a core patient right. Therefore, on-prem OSS deployments allow institutions to retain complete control over sensitive health data, ensuring that PHI never leaves local infrastructure. This transparency supports informed consent and strengthens patient trust. This is critical in domains such as oncology or psychiatry where confidentiality is paramount (71). In contrast, proprietary models often obscure data handling practices, creating tension with fairness and nondiscrimination mandates (61).

Explainability frameworks further bridge the gap between performance and compliance. By enabling clinicians to audit model behavior and validate outputs, explainability ensures that AI systems meet regulatory expectations for fairness and transparency. For instance, multilingual bias audits and human-in-the-loop validation help address disparities in diagnostic accuracy across patient populations, aligning AI with ethical governance principles. These strategies underscore the necessity of integrating explainability into clinical workflows to balance innovation with accountability.

In addition to these considerations, LLMs intended for clinical use may be classified as Software as a Medical Device (SaMD) and therefore require appropriate certification depending on their intended use and risk level. Such systems may fall under regulatory oversight frameworks including the U.S. Food and Drug Administration (FDA) and the European Union Medical Device Regulation (EU MDR). Clinical decision support generated by uncertified systems may not meet legal requirements for routine patient care. Notably, at least one commercially available LLM-based system (Prof. Valmed®) has obtained certification as a medical device (104), underscoring the growing regulatory scrutiny in this domain. Accordingly, compliance with SaMD regulations should be a central consideration when evaluating proprietary vs. open-source deployment strategies.

From a governance perspective, these deployment choices align with ethical and operational priorities. OSS models can support bias assessment (including subgroup-stratified performance checks) and explainability workflows in clinical AI systems. These practices align with emerging guidelines that emphasize fairness and transparency in healthcare AI (61). Proprietary systems, while delivering state-of-the-art performance, require compensatory safeguards such as contractual compliance clauses, external audits, and multilingual safety layers to mitigate risks associated with lack of transparency. Ultimately, the decision between OSS and proprietary models is not binary but contextual, depending on institutional priorities across five axes: performance (including schema fidelity), reproducibility, cost, transparency & auditability, and patient-centric governance.

## 4 Sustaining patient-centricity: emerging challenges in clinical AI deployment

Sustaining patient-centricity in clinical AI deployment presents new challenges, particularly in maintaining long-term engagement and adapting systems to evolving real-world contexts. Recent data show low patient trust in health systems' use of AI, underscoring the need for organizational strategies and transparent communication to support ongoing patient engagement (105). Adapting multilingual models to dynamic healthcare environments requires continuous refinement to address cultural nuances and language barriers, which may not be fully captured in static training data (106, 107). Regulatory changes and evolving ethical standards further complicate sustainability, necessitating flexible frameworks to align AI systems with shifting legal and societal expectations (106, 108). Additionally, scaling patient-centric benchmarks, such as interpretability and cultural sensitivity, across diverse populations demands cross-sector collaboration to integrate real-world data and patient-reported outcomes into training pipelines (109). Finally, balancing proprietary model performance with OSS transparency remains a challenge, as hybrid architectures must navigate trade-offs between innovation, accountability, and equitable access. Addressing these challenges requires embedding co-creation principles into technical and regulatory frameworks, ensuring AI innovations remain socially responsible and aligned with the changing needs of healthcare stakeholders.

## 5 Conclusion

The comparative analysis of open-source and proprietary LLMs in clinical IE underscores the nuanced trade-offs inherent in their deployment. OSS models excel in transparency, reproducibility, and local control, making them well-suited for environments prioritizing data sovereignty, auditability, and customization. Their flexibility allows for domain-specific adaptation, critical for addressing the heterogeneity of clinical data. However, they often require significant effort to achieve schema fidelity and robustness in complex tasks like FHIR-compliant data structuring. Proprietary models, while demonstrating superior performance in strict schema adherence and large-scale reasoning, face challenges related to opacity, cost, and regulatory compliance. Their black-box nature complicates bias auditing and raises concerns about data governance, particularly in privacy-sensitive settings.

Key challenges persist, including the need for standardized benchmarks, equitable performance across diverse patient populations, and the integration of patient-centric priorities such as fairness and explainability. The reliance on high-quality, representative training data remains a critical barrier, especially for rare diseases or underrepresented groups. To address this, hybrid approaches, combining the strengths of OSS preprocessing with proprietary generation, offer a pragmatic path forward, balancing technical capabilities with ethical considerations.

Ultimately, the choice between OSS and proprietary models must align with institutional priorities, including regulatory requirements, resource availability, and clinical objectives. Addressing these challenges requires sustained investment in data standardization, bias mitigation, and interdisciplinary collaboration. By prioritizing transparency, fairness, and adaptability, the healthcare community can apply LLMs to enhance clinical workflows while safeguarding patient trust and equity. The evolution of these technologies will depend on their ability to navigate technical, ethical, and operational complexities, ensuring their integration supports both innovation and responsible care. This analysis provides a structured foundation for understanding current trade-offs in local and proprietary LLM deployment within clinical information extraction. As with all rapidly evolving technologies, performance characteristics continue to evolve, and ongoing comparative evaluation remains essential to guide safe and effective clinical integration.
